# An Ishmaelite

**Published:** 1888-04-14

**Authors:** Leslie Keith

**Affiliations:** Author of "The Chilcotes," "Uncle Bob's Niece," "East and West," etc.


### An Ishmaelite.

By Leslie Keith,

Author of "The Chilcotes," "Uncle Bob's Niece," "East and West," etc.

### Chapter II.—Cousin Fanny appears on the scene.

That day, perhaps, decided Fred's relations with his father. For a long time the sound of his voice, heard suddenly, would set the boy trembling, and it was only after many threats and persuasions that he summoned courage to go with the others when the bell was rung on Sunday for the nursery folks to descend to dessert. How Fred stumbled through his portion of Church catechism he did not know, but he could not touch the nuts and raisins his mother heaped on his plate. It was all she could do to comfort her little son. On that dreadful night of his disgrace, when he was banished to the solitude of the empty room upstairs, he had lain sobbing in his pain and grief and terror, longing for his mother to come to him. But she did not dare to go. When Mr. Eastgate came upstairs, leaving the culprit below, after that administration of the rod, he merely said:

"The carriage is at the door, Helen; do you want your maid to fetch your wraps?" and when she opened her white lips in piteous appeal he cut her short sternly. "I won't have my son grow up a liar if I can help it," he said. "If there's no other way of teaching him the truth, I'll thrash it into him."

She submitted with a cowed resistance that only intensified her torture, since the resistance was without voice. She allowed her husband to wrap her mantle about her and lead her downstairs; she felt compelled to answer his remarks on the weather—on the company they were likely to meet. She had to take her place among the guests, and smile and talk as if she were happy, while her heart was bleeding. Even when they got home again she had to thrust back her yearning. As if he divined her secret intention, her husband followed her upstairs to her dressing-room.

"Helen," he said, while she stood with faltering fingers taking off her bracelets, "I know you are waiting till my back is turned to go up to the boy; but I forbid you to go. You have done your best to ruin him already, but I mean to put a stop to it. I mean to take him in my own hands."

"Oh, Joseph!" Her great trouble gave her courage, and she went up and put her hand tremblingly on his sleeve. "He is so little; he is only seven, and it is the first time——"

"It is not the first time. I have been questioning nurse——"

"Nurse is a wicked woman!" cried the outraged mother. "How does she dare to speak so of my child?"

"I have no reason to suspect *her* truthfulness," said Mr. Eastgate, coldly; "and when you call her a wicked woman, I dare say you forget that your grandmother recommended her. I understood that Lady Blackrock could do no wrong."

His sneer silenced her. She shrank before it.

"Look here, Helen," he said; and his voice was cool and determined, "if you shield the boy I shall find it out, and he and you will suffer for it. I've made up my mind. It isn't particularly pleasant to thrash my own son, but it's worse to have him deserve it by deceiving me; so you'd better leave the matter to me."

What could she do? She wept bitter tears that night when her husband slept. Her own forlorn childhood rose before her, as she thought of her little one sobbing his heart out in loneliness upstairs. "He is like me," she said; "my child is like me. If his father were gentle with him and helped him, he might grow up strong and brave; but harshness will make him a coward. Ah! why did I bring him into the world? His life will go wrong, and I shall live to see it, and it will be my punishment because I sold myself—because I married where I had no love to give." As she lay through the long silent hours she suffered far more than the child for whom her heart was aching. One may be weak and yet know it; perhaps the saddest thing of all is to be weak and know it, and yet be powerless to help it. Poor Helen was not blind to the fatal blemish in her own moral nature, and saw it with anguish repeated in her boy.

As he grew older he learned a little more self-control. He did not quake outwardly, though the inward fear remained. But it began to be no longer the fear of doing wrong, but the fear of being detected. He knew that he was not trusted; he knew that he was watched; his father's eyes seemed to pierce him when he asked him a question; he set lures to entrap the lad and catch him stumbling, and Fred grew cunning in evading them.

Something must be said for the father's point of view. The taint of insincerity that he discovered in his boy was a real shock to him. His own ostentatious, overbearing nature had never, in all its need of domination, felt the impulse to shield itself behind an untruth, and he had not sympathy enough to enter into any temptation that differed from his own. It was quite impossible for him to comprehend the nervous susceptibility—half of it physical—that made the boy shrink in dread of drawing down his father's displeasure, and catch at any chance that would save him from its consequences. Poor little lad! The father's austerity made a chill in his life that nothing but love could have melted. The love was there, perhaps, but it hid itself well, and the natural antipathy of temperament between the two was fast paving the way towards what might become hate.

"He despises me," thought the boy, half sullenly half in despair; and "What have I done to deserve a son who is a sneak and a coward," the father was daily asking himself, "while my brother, who is dead, leaves behind him a boy of whom any father might be proud?"

Little Arthur, a blue-eyed, chubby child, who was as open and artless as the day, was a constant wound to his uncle's pride. His very frankness seemed a reproach to Fred's timid reserve. He could scarcely bear him in his sight, and the child, who was much younger than his cousin, lived almost entirely in the upper regions, where his aunt would steal up to visit him. Sometimes she, too, suffered a jealous pang over this boy, who was stronger and manlier than her own child; but she was too good and kind-hearted to let it influence her.

When Fred was about twelve years old the question of school began to be discussed. Hitherto his education had been entrusted to a tutor whom the boy hated with all the strength of his small soul. He did not dare to rebel openly, but one day Nellie came upon him in the hall kicking furiously at the tutor's overcoat, which he had pulled down from its peg.

"I would serve him so if I could," he said, his sensitive face quivering with passion. "He's a sneak; he listens behind the door and watches me that he may tell papa and get me a thrashing."

"Oh," said Nellie in a melancholy voice, "I'm always being punished too. But it wouldn't be any good dancing on Miss Plumtree; she's so fat she wouldn't feel it. Come upstairs, Fred. I've got some chestnuts. Nursie has gone out with Arthur, and we'll roast them on the nursery fire. We'll lock the door so that old Miss Plumtree can't surprise us."

About this time cousin Fanny from the country managed to extort an invitation to spend a few weeks at Portland Place. The advance of spring brought round the necessity for her annual shopping once more, and it was much pleasanter to undertake the campaign of bargaining from a comfortable house than in poky lodgings, where you had to pay season's prices for everything.

The children watched this lady's arrival with a great deal of interest. She was big and dark, like uncle Joseph, and her voice was big and loud, like his, too. It came sounding up to that high region where the children were craning their necks over the banister, to the imminent risk of their lives.

"How do you do, Helen? *I* am quite well, for I never allow myself to think that I can be anything else. Take care of that box—the little one: I brought you some fresh eggs, Helen; all I could spare. Country eggs are so superior to town ones. I advise you to have one whipped up in your coffee every morning; you look as if you required making up."

"Oh, what a little box!" whispered Nellie, peeping between the rails. "Are country eggs very precious? And what has she got in that big box, I wonder?"

The big box was empty, but it was presently to be filled with the spoils of the London shops.

"I've come up for cheap bargains, Helen," she said in her big sounding voice. "The sales are a real godsend to poor clergymen with large families. If you don't like to come with me, you can let me have the carriage and I'll go alone."

Fanny always proclaimed her poverty for the information of everybody; it was the one thing about her that Joseph highly disapproved.

"We can't afford to be sensitive; I take all I can get," she said. And doubtless she knew that that little gift of country produce would be very handsomely repaid out of Helen's purse when it came to the shopping.

Joseph, however, if he disliked Fanny's parade of poverty as a reflection on his family, had in other respects a high opinion of her sense, and he proved it in a most gratifying manner by condescending on the very first evening to ask her advice about a school for Fred.

"I know the very thing for you," said Fanny promptly. She was one of the people who have a reserve for every case. "Not a school; a clergyman who takes pupils. He is a Balliol man, a good scholar, and a gentleman. His terms are high, but you wouldn't mind that."

"Of course not," said Joseph; "but what about the discipline? I can't trust the boy at a public school," he said bitterly. "He's not fit for it; he couldn't hold his own for a day."

"Oh, Mr. Jennings is strict enough; he has quite a reputation for breaking in refractory boys. There are those grandsons of Lord Vermillion—connections of yours, you know Helen—perfect little savages; he has brought them in wonderfully. And poor Lucy Chester's boy, whom she had half ruined with her injudicious indulgence, his guardian insisted on sending him to Mr. Jennings."

"Ah," said Mr. Eastgate bitterly, "if he cured him, he is the man for me."

Helen sat quivering and trembling with indignation and grief, but she was powerless to interfere. "Joseph never consulted me," she thought, sorely; "he would not listen to me now." And indeed, before half of Fanny's bargaining campaign was over, it was all settled; inquiries had been made and Fred's little outfit got ready.

"His tutor can't manage him," his father said when he was discussing these arrangements with Fanny; "the boy needs constant watching."

"Oh, Mr. Jennings will manage him," said Fanny with conviction. "No boy ever told *him* a lie twice."

It was only at the last that Helen took courage to make a timid appeal to Fanny.

"I hope Mr. Jennings will be kind to Fred," she said. "His papa doesn't understand him," she faltered; "he whips him."

"My dear Helen"—Cousin Fanny looked at her with round, black eyes, thinking what a very foolish little thing she was—"*every* boy needs to be whipped; we have never spared the rod with *ours*."

Helen was no doubt very feeble-minded. She never could speak at the right moment, and she was very easily silenced. She was silent now, and Fanny, who said a great many excellent things on the necessity of discipline and authority, and so forth, believed she had quite converted the little goose, and brought her to reason. But Helen's thoughts were bitter. "Fanny's boys, indeed! great, rough, clumsy creatures, who had no more feeling in them than a rhinoceros. They might be beaten indeed, without hurt; but her boy!"

Fred was, perhaps, the only member of the household who welcomed the impending change.

"I'm jolly glad to go," he said to the sad-hearted little Nellie, with whom he had shared so many seasons of disgrace. "It can't be worse than old Smythe, who is a beast."

"Oh, Fred," cried Nellie, opening her eyes, "if uncle heard you!"

"He can't," said Fred; but he cast a look of apprehension at the closed door. He drew nearer the little girl. "Mr. Smythe has gone away, Nellie; I saw him go, and I'm to have holidays till I go."

Nellie sobbed afresh. "Oh, Fred, I wish I was going with you!" she said. "I'm always in disgrace, and—and there will be nobody to tell it to when you are gone, except Aunt Helen; and she is always in disgrace, too."

It was natural that Fred, with the unquestioning hopefulness of childhood, should think that his life was going to be delightful because it was to be laid in new scenes, but there was to be no shifting of the background for Nellie. Miss Plumtree was not going, though Mr. Smythe had gone.

Nellie felt her on-coming desertion keenly. She made Fred a pen-wiper out of some fragments cut secretly from the inside of her new velvet frock, and she insisted on his accepting the half-crown which their joint godfather, Mr. Mack, had given her. Fred's half-crown, received on the same occasion from the old gentleman, had been long spent, but Nellie was glad she had kept hers.

The little woman had a very sad time of it during the last exciting days, when Fred's new possessions were coming home and his kit being packed, and she cried so much on the day of his departure that she made herself ill and was released from the school-room. When her aunt heard of it, she took the child to her own room and nursed her there tenderly, and the two Helens, the mother and the cousin, wept together for Fred.

*(To be continued.)*

